# Z-Ligustilide: A Potential Therapeutic Agent for Atherosclerosis Complicating Cerebrovascular Disease

**DOI:** 10.3390/biom14121623

**Published:** 2024-12-18

**Authors:** Longyu Shen, Qianqian Tian, Qiqi Ran, Qianrong Gan, Yu Hu, Donglian Du, Zehua Qin, Xinyi Duan, Xinyun Zhu, Wei Huang

**Affiliations:** 1 School of Basic Medical Sciences, Chengdu University of Traditional Chinese Medicine, Chengdu 611137, China; shenlongyu@stu.cdutcm.edu.cn (L.S.); qinzehua@stu.cdutcm.edu.cn (Z.Q.);; 2 Faculty of Social Sciences, The University of Hong Kong, Hong Kong 999077, China; 3 School of Clinical Medicine, Chengdu University of Traditional Chinese Medicine, Chengdu 611137, China; 20172025@cdutcm.edu.cn

**Keywords:** Z-ligustilide, atherosclerosis, ischemic cerebrovascular disease, intracranial atherosclerotic disease, ischemic stroke, transient ischemic attack, vascular dementia

## Abstract

Atherosclerosis (AS) is one of the major catalysts of ischemic cerebrovascular disease, and the death and disease burden from AS and its cerebrovascular complications are increasing. Z-ligustilide (Z-LIG) is a key active ingredient in *Angelica sinensis* (*Oliv.*) *Diels* and *Ligusticum chuanxiong Hort*. In this paper, we first introduced LIG’s physicochemical properties and pharmacokinetics. Then, we reviewed Z-LIG’s intervention and therapeutic mechanisms on AS and its cerebrovascular complications. The mechanisms of Z-LIG intervention in AS include improving lipid metabolism, antioxidant and anti-inflammatory effects, protecting vascular endothelium, and inhibiting vascular endothelial fibrosis, pathological thickening, and plaque calcification. In ischemic cerebrovascular diseases complicated by AS, Z-LIG exerts practical neuroprotective effects in ischemic stroke (IS), transient ischemic attack (TIA), and vascular dementia (VaD) through anti-neuroinflammatory, anti-oxidation, anti-neuronal apoptosis, protection of the blood-brain barrier, promotion of mitochondrial division and angiogenesis, improvement of cholinergic activity, inhibition of astrocyte proliferation, and endoplasmic reticulum stress. This paper aims to provide a basis for subsequent studies of Z-LIG in the prevention and treatment of AS and its cerebrovascular complications and, thus, to promote the development of interventional drugs for AS.

## 1. Introduction

Atherosclerosis (AS) is a gradual and lifelong process of accumulation and lesions of fatty and fibrous substances in the intima of blood vessels caused by various factors [1,2]. Secondary changes in later AS plaques result in restricted blood flow through the arterial lumen and trigger ischemic cerebrovascular diseases [3], including ischemic stroke (IS), transient ischemic attack (TIA), and vascular dementia (VaD). At present, cerebrovascular disease is the second leading cause of death and the third leading cause of death and disability combined worldwide [4,5]. AS remains an essential part of cerebrovascular disease, and along with the development of well-established risk factors for AS, such as diabetes mellitus, high cholesterol, and high BMI, the burden of disease caused by AS and its complications is rapidly and significantly increasing [1,6,7,8]. Therefore, intervention and prevention of AS, as well as treatment and control of AS complicating severe cerebrovascular disease, are necessary measures to address the remaining burden of AS and improve AS outcomes.

**Figure 1:**
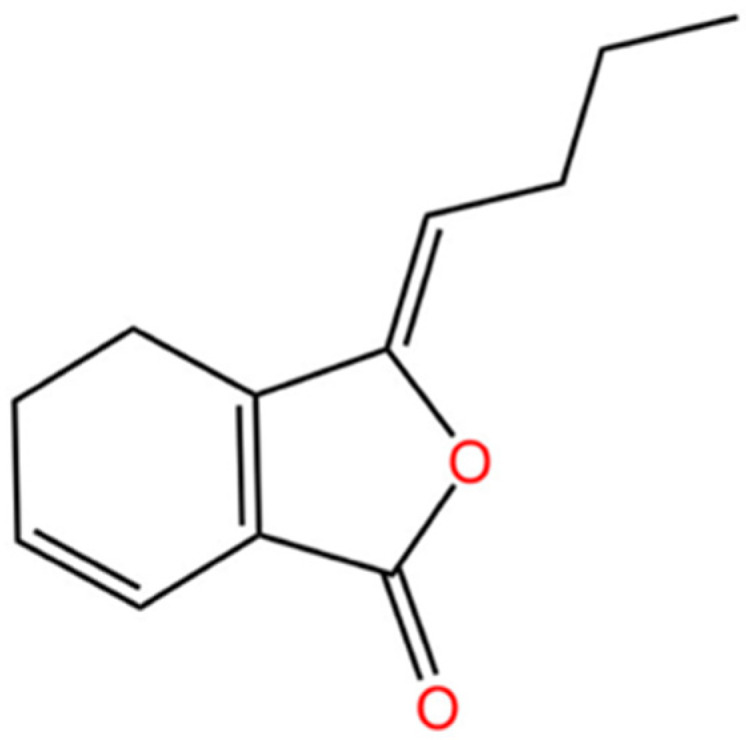
The chemical construction of Z-ligustilide.

Z-ligustilide (Z-LIG, PubChem CID: 5319022) is a characteristically active ingredient with high content in some medicinal plants of the family *Umbelliferae*, such as *Angelica sinensis* (*Oliv.*) *Diels* and *Ligusticum chuanxiong Hort* [9,10] (Figure 1). Modern pharmacological studies have found that Z-LIG has anticancer [11], anti-inflammatory [12,13], anti-apoptotic [14,15], and neuroprotective [16,17,18] effects. Preclinical studies have confirmed that Z-LIG also has a protective effect on the vascular endothelium [19]. It can effectively improve endothelial dysfunction and inhibit foam cell formation at the initiation of AS [19,20,21,22,23], preventing further vascular lesions. Z-LIG has also shown great potential in slowing down fibrous plaque formation and calcification during the development of AS [23,24,25,26,27,28]. When AS involves cerebral vasculature, Z-LIG plays a significant role in neuroprotective effects by reducing neuroinflammation [29,30], oxidative stress [30,31,32], and various other pharmacological activities. So far, there is a lack of studies that specifically collate pharmacological experiments on Z-LIG for treating AS and its cerebrovascular complications. Therefore, this paper collects pharmacological experiments of Z-LIG based on AS-related disease models and summarizes the preventive and therapeutic roles Z-LIG plays in AS-complicated cerebrovascular diseases.

## 2. Physicochemical Properties of Ligustilide

Ligustilide (LIG) is a light-yellow aromatic oil of unsaturated phthalides with the chemical formula C_12_H_14_O_2_ and a relative molecular mass of 190.24. LIG is soluble in organic solvents such as methanol, ether, and petroleum ether and has a boiling point of 168–169 °C. There is an extracircular double bond in the LIG structure, and therefore, there are two cis- and trans-isomers, i.e., Z-LIG and E-ligustilide (E-LIG) [33,34,35]. Z-LIG is less stable due to being a compound with reactive butenyl groups in three positions. It is susceptible to various reactions such as dehydrogenation, oxidation, and hydrolysis, resulting in isomerization to other phthalides with similar structures [35]. However, appropriately polar solvents, such as chloroform, can have a solvation effect on Z-LIG and thus effectively improve its stability for preparing and preserving Z-LIG [35,36]. Unlike in volatile oils, Z-LIG is vulnerable to degradation. Z-LIG in its pure form is susceptible to degradation and is more favorably preserved under sealing conditions, with protection from light, a low temperature of −20 °C, and a pH of 5.8–7.4. Compared to the Z-type, which has a spatially dominant conformation, the E-type is more unstable, and there is no practical way to improve its stability; thus, most LIG studies have chosen the Z-LIG structure, and this paper also discusses only the Z-type structure.

## 3. Pharmacokinetics of Ligustilide

Earlier studies suggest low bioavailability of orally administered Z-LIG. In rats administered orally at a dose of 500 mg/kg, the bioavailability was only 2.6%, which may be related to extensive metabolism in the liver [37,38]. However, in the oral route of administration, bioavailability does not necessarily correlate with dose [39]. By contrast, the pharmacokinetic properties of intraperitoneal Z-LIG show some non-linearity and dose dependence [37]. Available studies have demonstrated that nasal administration effectively avoids first-pass metabolism, has the advantages of quicker absorption and higher bioavailability, and can be used as a positive alternative to the traditional Z-LIG route of administration [40,41].

Z-LIG crosses the blood–brain barrier (BBB) and is characterized by highly selective distribution in the brain (particularly in the cerebellar region). The study revealed that at a dose of 20 mg/kg, Z-LIG from the oral route of administration in rats was distributed from the blood to the tissues at a moderate rate. The order of distribution of Z-LIG concentration in the tissues measured after 1.2 h was cerebellum > brain > heart (effector organ), spleen > kidney > liver (scavenging organ). The steady-state apparent volume of distribution (V) obtained after intravenous administration at doses of 12.5, 25, and 50 mg/kg was larger than the total water content, suggesting that Z-LIG is widely distributed in the rat [39].

Z-LIG exhibits rapid metabolism and high clearance in both rat and human hepatocytes [42]. However, in vivo experiments show that the clearance of Z-LIG gradually slows down within 3–12 h after oral administration in the rat model, which may be connected with the binding of Z-LIG to serum proteins, leading to drug accumulation [38]. Z-LIG is cleared in vivo at a rapidly increasing rate after intravenous administration, and the total body plasma clearance is significantly higher than the cardiac output reported in blood-based rats. Z-LIG also appears to undergo first-pass effects in the rat lungs and heart, although the mechanism is uncertain yet [39]. In addition, the intraventricular clearance of Z-LIG extracted from *Ligusticum chuanxiong Hort* is significantly higher than that of its pure form under the intravenous administration route, suggesting a significant interaction between Z-LIG and the components in the extract [37]. Furthermore, CYP3A4, CYP2C9, and CYP1A2 are the primary metabolic catalases of Z-LIG [43]. The metabolic pathways of Z-LIG include epoxidation, epoxide hydrolysis, aromatization, hydroxylation, and glutathionylation [42,44]. Twenty-four metabolites of Z-LIG have been identified [37,42,43,44,45], including senkyunolide I, 6,7-epoxyligustilide, and others [21,37,42,43,44]. In urine, Z-LIG is eliminated mainly as a hydroxylated product, and only a tiny amount of Z-LIG is metabolized in the intestine [37,44].

## 4. Pathogenesis of Atherosclerosis

AS typically begins with endothelial dysfunction caused by recurrent, chronic injury to the vascular endothelium. The pathological state of the vessel wall is prone to events that induce an inflammatory response, such as lipid infiltration, monocyte adhesion, platelet activation, and oxidative stress, which is considered to be the beginning of fatty streak formation in the early stages of atherosclerotic plaques [46]. In general, endothelial cells (ECs) reject monocytes adhering to their surface, but activated ECs overexpress cell adhesion molecules (CAMs) to induce monocytes recruitment to the vascular intima [47]. Parallel changes in endothelial permeability and extracellular matrix (ECM) composition can promote low-density lipoprotein (LDL) entry and retention in the subendothelium [48]. Under pathological conditions, LDL particles that have been oxidized or otherwise modified accumulate in the vascular intima and induce monocytes to enter [49,50]. Monocytes differentiate in the intima into macrophages capable of expressing high-capacity scavenger receptors for LDL particles, which allows macrophages to overload lipoprotein particles and become atherosclerotic plaque precursors, foam cells [48].

Vascular smooth muscle cells (VSMCs) are also a major source of AS plaques and ECM [51,52]. That process contributes to AS progression through complex interactions between multiple mechanisms such as lipid retention, oxidation, inflammation induction, proliferation of VSMCs, and phenotypic switching [53]. After a large number of foam cells form the fatty streak, multiple chemokines and growth factors produced by activated ECs and macrophages induce the proliferation of neighboring smooth muscle cells (SMCs) within the endothelium, as well as the synthesis of collagen, proteoglycans, and other ECM components, and collagen fibrils secreted by the SMCs cause the fatty streak to slowly fibrillate, thus transforming it into a “fibrous plaque”, which is followed by the formation of a fibrous cap [54,55]. The fibrous cap typically covers a collection of macrophage-derived foam cells, and the low-efficiency process of removing dead cells from them can lead to the accumulation of cellular debris and extracellular lipids to form a lipid-rich “lipid pool” known as the necrotic core of the plaque [56]. Calcification, a marker of advanced plaques, then extends from the base of the necrotic core into the surrounding matrix in a bone-like form within the plaque [57].

**Figure 2:**
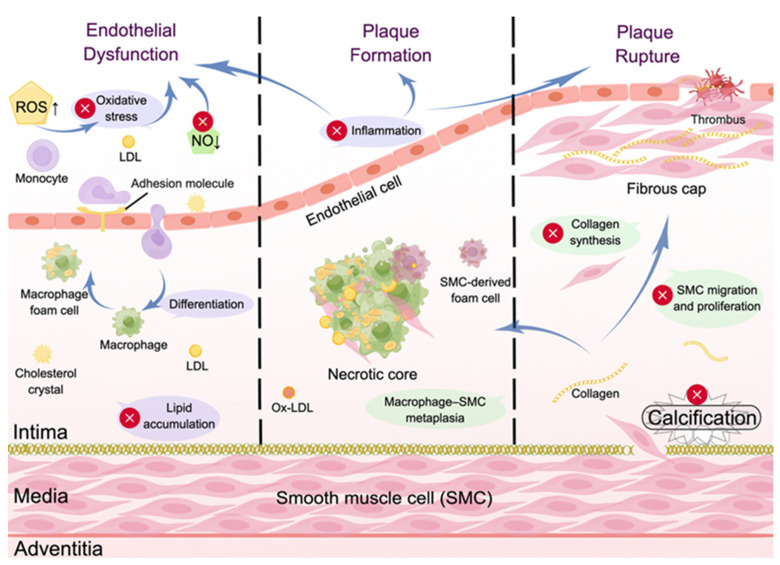
Z-ligustilide intervention in different links of atherosclerotic lesions. Z-ligustilide improves endothelial dysfunction before initiating atherosclerotic lesions and counteracts the development and rupture of atherosclerotic plaques. The picture is generated by Figdraw v.1.0.

Plaque rupture and erosion are considered triggers for dangerous lesions in the more advanced stages of AS [3]. Factors such as matrix remodeling, microscopic calcification within the necrotic core and fibrous cap, and the inflammatory response induce plaque rupture [3,54,58,59,60]. Plaque rupture promotes thrombosis, and plaque healing activates thrombolysis, granulation tissue formation, and vascular reendothelialization, although thrombosis stabilizes the plaque [61]. However, the repetitive process of plaque rupture and healing exacerbates the growth of the lesion volume, and eventually, the plaque invades the arterial lumen, leading to persistent and occlusive thrombosis [62]. Unlike typical ruptured plaques, eroded plaques do not have a thin and fragile fibrous cap but possess a more abundant ECM and a small number of inflammatory cells [63,64]. In addition, they account for an increasing proportion of acute episodes of thrombotic complications of AS [3]. Thus, AS, a systemic disease involving multiple blood vessels [3], is typically caused by tissue ischemia due to plaque restriction of blood flow or local disruption of blood flow resulting from thrombus or embolic retention. This results in an acute or sudden clinical presentation that leads to severe complications [48] (Figure 2).

## 5. Atherosclerosis Complicating Cerebrovascular Disease

Intracranial atherosclerotic disease (ICAD) is a common cerebrovascular complication of AS, including IS and TIA [3,65,66]. Although, unlike an ischemic stroke, transient ischemic attacks do not generally result in significant infarction [67], both IS and TIA can initiate an ischemic cascade and ultimately mediate secondary neurodegenerative pathology [68,69]. Therefore, IS and TIA are considered essential factors in the pathogenesis of VaD [70,71,72,73,74,75]. Dementia is a neurodegenerative disease that interferes with the functioning of daily activities [71,76]. Vascular cognitive impairment (VCI) is one of the most common types of dementia. When the concept of VCI was widely accepted, vascular dementia (VaD) was considered the most severe form of VCI [72,77,78,79]. In addition, there is growing evidence that AS and VaD share some common risk factors and pathogenic pathways. Hence, reducing the impact of vascular lesions on VaD is a promising strategy for reducing future risk [80,81].

## 6. Mechanisms of Action of Z-Ligustilide Intervention in Atherosclerosis

### 6.1. Improvement of Endothelial Dysfunction and Inhibition of Fatty Streak Formation

#### 6.1.1. Improvement of Lipid Metabolism

Triglycerides (TG) are predominantly found in triglyceride-rich lipoproteins (TRLs), and evidence from genetic studies suggests that high levels of TRLs are causally associated with AS and that a reduction in blood TRL concentrations can help mitigate AS and inflammation [82]. In addition, prolonged and cumulative exposure to low-density lipoprotein cholesterol (LDL-C) in the arteries has been recognized as a critical link in the development of AS, which drives the further development of cardiovascular diseases and its primary clinical sequelae [83]. Z-LIG can potentially ameliorate dyslipidemia and exert anti-AS effects at the link of lipid infiltration.

Fatty acid transporter protease 5 (FATP5) promotes bile acid reactivation, hepatic free fatty acid uptake, and lipid accumulation. Knockdown of FATP5 in vivo prevents and reverses fatty liver in mice [84]. Diglyceride acyltransferase 2 (DGAT2) down-regulation reduces fatty acid synthesis and inhibits hepatic TG secretion and accumulation [85]. Z-LIG mediates the effects on fatty acid uptake and esterification in HepG2 cells by down-regulating the expression of FATP5 and DGAT2 and effectively inhibits oleic acid-induced lipid accumulation in a dose-dependent manner [20].

Z-LIG can inhibit the biotransformation of HMG-CoA by irreversible covalent binding of its metabolic intermediate 6,7-epoxyligustilide (EM-Lig) to Cys129 in 3-hydroxy-3-methylglutaryl coenzyme A synthetase 1 (HMGCS1). Thus, Z-LIG lowers plasma LDL-C and total cholesterol (TC) levels to perform anti-AS effects. HMGCS1 is a critical enzyme in the mevalonate pathway of hepatocyte cholesterol synthesis. Zhang et al. investigated the lipid-lowering effects of Z-LIG from a chemical proteomics perspective by designing an alkynyl-modified Lig (AM-Lig). They found that Z-LIG metabolized to EM-Lig could reduce HMGCS1 enzyme activity by attacking Cys129 of HMGCS1 to block its acetylation, thereby reducing cholesterol biosynthesis and ameliorating dyslipidemia [21].

#### 6.1.2. Anti-Oxidation

One crucial mechanism in the formation of AS is the generation of excessive reactive oxygen species (ROS) in response to oxidative stress, which is an essential cause of endothelial dysfunction [86,87]. ROS induces the expression of scavenger receptors in SMCs to promote their transformation to foam cells. ROS also causes the release of matrix metalloproteinases (MMPs), which degrade the fibrous wall of AS plaques and the basement membrane of ECs and promote the proliferation of smooth muscle and the accumulation of collagen, contributing to the disruption and transformation of AS plaques [88]. Zhu et al. revealed that Z-LIG can activate nuclear factor erythroid 2-related factor 2 (Nrf2) in EA.hy926 cells to upregulate the expression of various antioxidant genes and inhibit ROS production, thereby protecting the vascular endothelium from oxidative stress. Z-LIG preconditioning counteracted decreased reduced glutathione (GSH) content and increased glutathione disulfide (GSSG) levels under tert-butyl hydroperoxide (t-BHP) treatment. Meanwhile, they demonstrated that Z-LIG upregulated the expression of Nrf2 and ARE kinesin in a dose- and time-dependent way and promoted the accumulation of nuclear Nrf2 and unbound Nrf2-Keap1 complexes, suggesting that the attenuation of t-BHP-induced cellular injury correlates with the activation of the Nrf2/ARE pathway. In addition, Z-LIG may significantly reduce lipid levels, inhibit lipid peroxidation, and stimulate antioxidant enzyme activities in mice through activation of Nrf2 and ARE driver genes, indicating that Z-LIG effectively prevents endothelial damage in HFD-fed Ldlr^−/−^ mice [22].

#### 6.1.3. Anti-Inflammatory

The inflammatory process occurs throughout the entire life of AS, from endothelial activation injury to plaque rupture and healing [89,90]. As a natural anti-inflammatory substance, Z-LIG can regulate cytokine- and chemokine-dependent inflammatory responses while exerting anti-inflammatory activity [91,92,93,94,95]. Z-LIG has been proven to inhibit inflammation-mediated monocyte adhesion and migration to the intima and the calcification of subsequent AS plaques during the progression of AS [19,23].

Activated ECs express CAMs to mediate blood monocytes to complete rolling, adhesion, and access to the intima, thereby accelerating macrophage-derived foam cell formation [96,97,98]. CAMs can be expressed through ROS/NF-κB signaling initiated by the inflammatory cytokine TNF-α (tumor necrosis factor alpha) from endothelial immune cells [99]. Chio et al. demonstrated that Z-LIG effectively inhibited the expression of cellular CAMs, including vascular cell adhesion molecule-1 (VCAM-1), intercellular adhesion molecule-1 (ICAM-1), and endothelial-selectin (E-selectin). Further studies revealed that Z-LIG attenuated TNF-α-stimulated adhesion of HL-60 monocytes in human umbilical vein endothelial cells (HUVECs) through suppression of ROS/NF-κB signaling [19]. Thus, it is likely that Z-LIG mediates anti-AS efficacy by attenuating chronic inflammation in the vascular endothelium.

#### 6.1.4. Endothelial Protective Effect

Endothelial dysfunction is intimately associated with a decrease in nitric oxide (NO) bioavailability. Z-LIG upregulates the expression of NO and heme oxygenase 1 (HO-1), which have vasoprotective effects [98,100]. As an AS-protective molecule, NO maintains metabolic homeostasis with endothelium-dependent vasodilation, inhibiting pro-inflammatory cytokine secretion, SMCs proliferation, and thrombosis [100,101,102]. Similarly, HO-1 expression in ECs has been demonstrated to attenuate AS [103]. Chio et al. revealed that Z-LIG significantly regulated NO and HO-1 expression in HUVECs in a dose-dependent manner and that the induction of HO-1 expression by Z-LIG may be relevant to Nrf2 nuclear translocation and NO synthesis [19].

### 6.2. Inhibition of Fibrous Plaque Formation and Calcification

#### 6.2.1. Alleviation of Vascular Endothelial Fibrosis

Vascular fibrosis, characterized by excessive ECM accumulation, contributes to the decline in vascular function, and Z-LIG may exert an anti-AS effect by alleviating vascular endothelial fibrosis [104]. Vascular fibrosis transforming growth factor-β (TGF-β) is a potent inducer of ECM and upregulates ECM proliferation and accumulation and vascular remodeling [105]. The TGF-β signaling mediates most fibrotic processes, and SMAD3, a signal transducer of TGF-β, synthesizes proteoglycans and collagen. Dysregulation and sustained activation of TGF-β/Smad3 signaling plays a crucial role in initiating and maintaining the fibrotic tissue phenotype [106]. Lei et al. chose a chemical proteomics approach using a photoaffinity-labeled probe (Lig-PAL) of the active ingredient of Z-LIG, targeting SMAD3. The Lig-PAL probe demonstrated that Z-LIG blocked SMAD3 binding to TGF-β receptor 1 to inhibit the phosphorylation and nuclear translocation of SMAD3 to impede collagen synthesis in the course of AS, thereby attenuating vascular endothelial fibrosis. Moreover, Z-LIG effectively inhibited the TNF-α-induced expression of collagen I (COL I) and collagen III (COL III) in mouse aortic endothelial cells (MAECs) [28].

#### 6.2.2. Inhibition of the Pathological Thickening of the Vascular Endothelium

The excessive proliferation, migration, and recruitment of VSMCs create many extracellular matrix molecules that contribute to pathological vascular intimal thickening, which is conducive to the growth and development of AS plaques [107]. Z-LIG plays an obstructive role in this process. Earlier studies have identified that Z-LIG can significantly down-regulate the MAPK pathway to inhibit the proliferation and cell cycle progression of VSMCs by reducing the production of ROS [24]. C-Myc is an essential mediator of cell growth and development and cell migration. Activated c-Myc promotes the transformation of SMCs from contractile to secretory and induces overexpression of MMPs to accelerate cell migration and ECM breakdown [108]. Deng et al. demonstrated that Z-LIG down-regulated the c-Myc pathway and inhibited the migration and phenotypic switching of VSMCs to ameliorate pathological endothelial thickening induced by AS in spontaneously hypertensive rats [25]. Rho-associated kinase (ROCK) is an important effector molecule downstream of RhoA that activates c-Jun N-terminal kinase (JNK) to mediate cell contraction. Thus, ROCK plays an essential role in vascular endothelial cell migration and vascular remodeling [109]. Luo et al. found that Z-LIG could inhibit the migration of VSMCs induced by angiotensin II (AngII) by downregulating the expression of c-Myc/MMP2 and ROCK/JNK signaling pathways. In addition, this is the first study to find that intraperitoneal injection of Z-LIG can also inhibit pathological endothelial thickening [26].

Recent studies have shown that VSMCs autophagy plays a vital role in inhibiting AngII-mediated proliferation and migration of VSMCs [110]. It was found that Z-LIG also inhibited AngII-induced autophagic flux in A7r5 cells through activation of the Akt/mTOR signaling pathway and suppressed the development of pathological changes such as endothelial thickening caused by phenotypic switching in VSMCs [27].

#### 6.2.3. Inhibition of Plaque Calcification

Z-LIG decelerates plaque calcification by inhibiting AS immune and inflammatory responses. Vascular cells may undergo chondrogenic or osteogenic differentiation, resulting in membranous bone mineralization and endochondral bone formation. Particularly in endochondral calcification, calcified vascular cells are more likely derived from local SMCs and circulating hematopoietic stem cells [111]. CD137 (4-1BB) is a membrane surface protein belonging to the tumor necrosis factor receptor superfamily (TNFRSF), which promotes the development of human AS plaques and decreases the instability of advanced AS plaques. In addition, protein kinase B (Akt) participates in the transcription and overexpression of CD137 [112]. Activation of CD137 signaling accelerates vascular calcification in vivo and promotes the calcification of VSMCs in vitro, and this effect may be relevant to stimulating osteogenic differentiation of VSMCs [113]. Transcriptional activation of CD137 requires recruitment of NF-κB and activator protein-1 (AP-1) to its promoter site [114]. Experimental evidence showed that Z-LIG down-regulated AP-1 expression and effectively reduced the expression of CD137, a diagnostic marker of AS plaque instability, potentially through inhibition of AP-1 and the Akt/NF-κB signaling pathway [23] (Table 1).

## 7. Mechanisms of Z-Ligustilide in the Treatment of Cerebrovascular Complications in Atherosclerosis

### 7.1. Z-Ligustilide and Intracranial Atherosclerotic Disease

#### 7.1.1. Anti-Neuroinflammatory Effects

The sterile inflammatory response due to ischemia-reperfusion involves signaling by pattern recognition molecules such as Toll-like receptors (TLRs) [69]. TLR4 has been the most widely studied in the TLRs. It recognizes damage-associated molecular patterns (DAMPs) released from ischemia-induced sterile damaged cells, and oxidative stress due to ischemia-reperfusion enhances TLR4 activation [115,116]. DAMPs may function as endogenous ligands for TLR4, mediating the TLR4/NF-κB signaling pathway to enhance innate and adaptive immunity, thereby promoting post-ischemic neuroinflammation and cerebral infarction [69,117]. The extracellular release of peroxiredoxins (Prxs) during the acute and subacute phases of ischemic stroke activates TLR4, exacerbating the neuroinflammatory response and brain damage. Thus, Prx blockers may serve as an effective neuroprotective tool in ischemic stroke [118]. The Prx family has six isoforms, Prx1–Prx6. Prx6 is a newly identified TLR4-dependent inducer abundantly expressed in astrocytes and may be an essential target for immune regulation and neuroinflammation after ischemic stroke [118,119]. Post-ischemic treatment with Z-LIG significantly reduced TLR4 expression and ERK 1/2 phosphorylation, inhibited NF-κB signaling, and up-regulated Prx5 and Prx6 expression. This suggests that Z-LIG attenuates neuroinflammatory responses after cerebral ischemia-reperfusion through down-regulation of the TLR4/Prx6 signaling pathway and has a direct neuroprotective effect against ischemic brain injury. In addition, Treg cells are considered one of the key immunoprotective regulators in the ischemic cascade [120,121]. Z-LIG appears to regulate Treg cell number and function [29]. It has been reported that Prxs have significant translational potential for known DAMPs [122,123]. Zhao and colleagues further explored the effect of Z-LIG on the inflammatory response of members of Prxs in macrophages based on the experiments of Kuang et al. They found that Z-LIG treatment effectively inhibited the production of pro-inflammatory mediators in macrophages induced by Prx1, Prx2, and Prx4, as well as the activation of the TLR4/NF-κB pathway [124].

Astrocytes and microglia play an essential role in the pathogenesis of various ischemic neurodegenerative diseases, and blocking microglia activation to reduce the production of a wide range of mediators can inhibit the neuroinflammatory response. Z-LIG significantly inhibited the neuroinflammatory toxicity induced by microglia activation [125,126]. Lipopolysaccharide (LPS), a natural TLR4 ligand that initiates NF-κB pathway signaling, is widely used to activate microglia [117,127]. The in vitro experiments designed by Wang et al. revealed that Z-LIG effectively inhibited the inflammatory response resulting from LPS-induced activation of primary rat microglia in a concentration-dependent manner and that this effect also appeared to be mediated by affecting the NF-κB signaling pathway [128].

RIG-I has been reported to act as a positive regulator of NF-κB signaling [129]. Similarly, Z-LIG is likely to block the development of neurological deficits and reduce neuronal loss in the cornu ammonis 1 (CA1) and caudate-putamen (CPu) regions after cerebral ischemia by inhibiting the RIG-1/NF-κB p65 pathway [30]. The Klotho (KL) protein has been demonstrated to act as a neuroprotective factor by inhibiting inflammation and oxidative stress [130,131]. Further, the anti-inflammatory effect of *KL* is associated with inhibiting the RIG-I-mediated transcription factor NF-κB [132]. After the down-regulation of *KL*, the protective effect of Z-LIG on neurons in the CA1 and CPu regions of bilateral common carotid occlusion (BCCAO) mice was relatively attenuated [30], suggesting that *KL* is involved in the protection of Z-LIG against cerebral ischemic injury in mice.

#### 7.1.2. Anti-Oxidation

Oxidative stress produced by ischemia-reperfusion stimulates increased responsiveness of inflammatory cells to subsequent stimuli [133]. Over the past few years, an enormous amount of evidence has suggested that oxidative stress associated with the overproduction of ROS is the underlying mechanism of brain damage in stroke and reperfusion secondary to stroke. Excess ROS can be partially scavenged by endogenous antioxidants, such as superoxide dismutase (SOD), glutathione peroxidase (GSH-Px), and catalase (CAT), among others [134,135]. Kuang et al. first proved that treatment with Z-LIG after cerebral ischemia-reperfusion significantly reduced the volume of cerebral infarction in transient forebrain cerebral ischemia (FCI) mice while restoring brain tissue GSH-Px and SOD activities, which indicated that Z-LIG markedly attenuates lipid peroxidation in the brain tissue of FCI mice [32]. Z-LIG not only triggers the stress response by inducing ROS formation and transient depletion of GSH but also activates survival-promoting signals through the cross-talk of the PI3K and Nrf2 pathways; specifically, Z-LIG induces HO-1 protein expression and nuclear translocation of Nrf2 in a concentration- and time-dependent manner and induces Akt kinase and GSK3b phosphorylation to protect PC12 cells from oxygen–glucose deprivation (OGD)-induced oxidative stress. Further studies revealed that this protective effect may be related to the interaction between the PI3K/Akt pathway and the Nrf2/HO-1 pathway [31]. Furthermore, activation of Akt leads to the inactivation of forkhead box class O (FoxO), which can mediate ischemic tolerance after hypoxic preconditioning [136]. Z-LIG was identified to exert antioxidant effects through up-regulation of the transcription factor FoxO1, suggesting that the anti-oxidative stress effects of Z-LIG in ischemia may also relate to inhibition of the Akt/FoxO1 pathway [30].

#### 7.1.3. Anti-Neuronal Apoptosis

As a form of regulated and programmed cell death, apoptosis plays an essential role in cerebral ischemia-induced brain injury in humans and animal models [137,138]. It allows initiation by disrupting mitochondrial functional homeostasis and releasing the pro-apoptotic protein cytochrome c (Cytc) or other apoptosis-inducing factors such as the caspase family [139,140,141]. The Bcl-2 family can regulate apoptosis and necrosis. The pro-apoptotic protein Bax and the anti-apoptotic protein Bcl-2 play critical regulatory roles in the mitochondrial pathway as vital members of the Bcl-2 family. The ratio of Bax/Bcl-2 is an important indicator related to apoptosis [142,143]. Z-LIG may inhibit the neuron-associated caspase-3 apoptotic pathway. Earlier experiments by Kuang et al. found that Z-LIG treatment significantly up-regulated the expression of Bcl-2 in forebrain ischemia model mice while significantly down-regulating the expression of Bax and caspase-3 in ischemic brain tissue [32]. Wu et al. demonstrated that Z-LIG activated the PI3K/Akt signaling pathway to reverse hippocampal neuronal apoptosis caused by ischemia-reperfusion (I/R). In cultured primary hippocampal neurons under oxygen–glucose deprivation/reperfusion (OGD/R) and the middle cerebral artery occlusion and reperfusion (MCAO/R) rat model, Z-LIG attenuated neuronal injury, down-regulated Ca^2+^ influx and ROS production in hippocampal neurons, significantly inhibited Cytc release, and decreased caspase-3 expression and the Bax/Bcl-2 ratio. Further experiments demonstrated that Z-LIG inhibited OGD/R-induced apoptosis associated with activation of the PI3K/Akt signaling pathway [144].

Autophagy-mediated cellular outcomes are dualistic; autophagy generally reduces cellular damage by blocking apoptosis and inhibiting the activation of apoptosis-associated caspases [145]. Thus, autophagy is considered a survival-promoting pathway in the central nervous system (CNS) that protects cells from cerebral ischemic injury, and a certain degree of enhanced autophagy can help reduce neuronal damage [146,147]. Beclin1 regulates other autophagy proteins and reduces the accumulation of LC3-II [148]. Besides, the mTOR is a vital regulator of stroke-related autophagy [149]. AMPK acts on mTOR kinases and upregulates autophagy through phosphorylated activation of several upstream kinases, including liver kinase B1 (LKB1) [150,151]. Zhao et al. explored whether Z-LIG could inhibit apoptosis via autophagy by studying its effect on autophagy in PC12 cells exposed to OGD/R. The experimental results revealed that Z-LIG treatment promoted elevated LC3-II/LC3-I ratio, Beclin1 protein expression, and autophagosome formation in OGD/R PC12 cells. Furthermore, elucidating the relationship between the LKB1-AMPK-mTOR signaling pathway and apoptosis by the autophagy inhibitor 3-MA or the AMPK inhibitor dorsomorphin, they found that Z-LIG was able to promote autophagy and protect PC12 cells from OGD/R-induced apoptosis via the LKB1-AMPK-mTOR signaling pathway [152].

#### 7.1.4. Protective Effect of the Blood–Brain Barrier

Disruption of BBB integrity is one of the crucial mechanisms of neuronal dysfunction and death after cerebral ischemia, and protection of the BBB somewhat improves post-ischemic stroke outcomes [153]. Wu et al. explored the effect of Z-LIG on BBB permeability and its mechanism by OGD-induced ischemic injury in the model of rat brain microvascular endothelial cells co-cultured with astrocytes. Their study demonstrated that Z-LIG could reduce OGD-induced permeability in an in vitro co-culture BBB model, which appeared to be achieved by protecting the tight junctions (TJs) inhibition and HIF-1a/VEGF pathway [154]. TJs are critical structural components of the BBB and are directly related to BBB integrity [155]. Vascular endothelial-derived growth factor (VEGF) is an essential protein of hypoxia-inducible factor 1 (HIF-1) that induces microvascular leakage [156]. Aquaporin-4 (AQP-4), one of the major water channel proteins widely distributed in the brain, significantly promotes cerebral edema after cerebral ischemia, and there is a causal relationship between it and BBB integrity [157]. OGD injury induced the expression of HIF-1α, which up-regulated AQP-4 and VEGF and caused the degradation of the basement membrane and the tight junction proteins zonula occludens-1 (ZO-1) and occludin, leading to an increase in permeability and the destruction of the BBB. Z-LIG was able to reverse the down-regulation of OGD-induced expression of ZO-1 and occludin. Meanwhile, Z-LIG effectively reduced BBB permeability by blocking the HIF-1a/VEGF pathway [154]. Li et al. also demonstrated that Z-LIG up-regulated the expression of ZO-1 and occludin and protected the structure and function of TJs to a certain extent. In addition, they found that intranasal Z-LIG pretreatment effectively inhibited the increase of matrix metalloproteinase-2 (MMP-2) and matrix metalloproteinase-9 (MMP-9) levels after ischemic injury and restored the loss of collagen IV induced by cerebral ischemic injury, thus reducing the loss of ECM components of the basement membrane and the disruption of the stability of the BBB [158].

#### 7.1.5. Promotion of Mitochondrial Division

PINK1/Parkin has been reported to be a classical pathway mediating mitochondrial autophagy in CNS diseases [159]. PINK1 stably accumulates on the outer membrane of damaged mitochondria under ischemic conditions. It recruits Parkin to initiate mitochondrial autophagy, which removes dysfunctional mitochondria in a timely and selective manner, thus exerting neuroprotective effects [160,161,162,163,164,165]. Mao et al. found that Z-LIG significantly promoted mitochondrial autophagy, and this effect was blocked by PINK1 deficiency and mitochondrial division inhibitor 1 (Mdivi-1) under cerebral ischemia-reperfusion injury (CIRI) conditions, suggesting that Z-LIG enhanced PINK1/Parkin pathway-mediated mitochondrial autophagy to attenuate cerebral ischemia-reperfusion-induced neuronal injury [166]. Mitochondrial division is a prerequisite for mitochondrial autophagy, and exacerbation of ischemic injury may be associated with inhibition or down-regulation of dynamin-related protein 1 (Drp1), a fundamental fission regulator [167]. As a critical sensor of cellular energy status, AMPK can recruit Drp1 to the mitochondrial membrane and activate mitochondrial division by signaling mitochondrial fission factor (Mff) [168]. Wu et al. focused more on exploring the relationship between Z-LIG-induced mitochondrial division and mitochondrial autophagy when investigating the role of Z-LIG in improving mitochondrial function in ischemic stroke, and their results suggest that Z-LIG induces Drp1-mediated mitochondrial division and improves mitochondrial function in an AMPK-pathway-dependent manner, which in turn attenuates ischemic stroke injury [169].

#### 7.1.6. Promotion of Angiogenesis

Z-LIG promotes focal angiogenesis after ischemia. Angiogenesis is a crucial recovery mechanism in response to ischemia, and it is involved in brain plasticity and functional recovery after stroke. Potential mechanisms to improve angiogenesis and remodeling could be beneficial in achieving more effective treatment of ischemic stroke [170,171]. Improvement in neurological function with Z-LIG correlates with increased cerebral vascular density, and Z-LIG treatment facilitates increased cerebral microvessel density and improved neurological function in MCAO mice. Z-LIG elevated the tube-forming capacity of mouse brain endothelial cell line (bEnd.3) cells, including the promotion of proliferation, migration, and lumen formation of bEnd.3, suggesting that Z-LIG enhances angiogenesis in both in vitro and in vivo ischemic models. In addition, VEGF expression and endothelial nitric oxide synthase (eNOS) activation were increased in the ischemic hemisphere of mice after Z-LIG treatment, demonstrating that the neuroprotective effect of Z-LIG is partially attributable to the enhancement of the local VEGF/eNOS pathway [172]. The VEGF/eNOS pathway is tightly associated with ischemic neovascularization [173,174]. VEGF stimulates NO release from vascular endothelial cells by enhancing eNOS expression and phosphorylation, thereby inducing endothelial cell proliferation and migration and promoting angiogenesis [174].

#### 7.1.7. Other Mechanisms

Z-LIG promotes the transcription of the endogenous protective factor erythropoietin (EPO) and downregulates the expression of the endogenous deleterious factor RTP801 to mediate neuroprotection against ischemic injury. EPO is a glycoprotein hormone that primarily regulates erythropoiesis in response to hypoxia [175]. The erythropoietin receptor (EpoR) is widely expressed in a wide range of brain cells. Combining the two activates vital signaling pathways such as cellular differentiation, apoptosis control, and neuroprotection, thus exerting significant regulatory effects [176,177]. HIF-1α promotes increased transcription of EPO in ischemic injury [178,179]. The stress-inducible protein RTP801, the product of a cloned gene capable of being strongly upregulated by hypoxia both in vitro and in vivo, appears to contribute to apoptosis under certain circumstances [180]. Experiments by Wu et al. investigated the effects of Z-LIG on EPO and RTP801 expression in I/R rats and whether these effects were related to the neuroprotective effects of Z-LIG. They found that pretreatment with Z-LIG reduced neurological deficit scores and infarct volume in I/R rats in a dose-dependent manner and increased cell viability of neurons exposed to OGD in vitro, suggesting that EPO is one of the crucial mediators of the neuroprotective effects of Z-LIG. Z-LIG promotes the phosphorylation of ERK and thus upregulates the phosphorylation of HIF-1. This change increases the trans-activating activity of HIF-1 and finally upregulates EPO expression, suggesting that Z-LIG promotes EPO transcription through the ERK signaling pathway. Meanwhile, the neuroprotective effect of Z-LIG may also be correlated with the inhibition of RTP801 expression [181].

Enhanced ischemic tolerance and preventive effects of Z-LIG on cerebral ischemic injury are associated with Nrf2 and heat shock protein 70 (HSP70). Nrf2 and stress-induced HSP70 have positively regulated multiple mechanisms involved in the ischemic cascade response, including oxidative stress, neuroinflammation, and proteotoxic stress [182]. Overexpression of HSP70 in hippocampal CA1 neurons after reperfusion of middle cerebral artery occlusion reduced protein aggregation, including degradation of unstable and misfolded proteins and translocation of proteins between cellular compartments, contributing to neuronal survival [183,184,185]. Z-LIG induced protective HSP70 expression both in vivo and in vitro, which appeared to be associated with transient activation of MAPK. Whereas both Nrf2-mediated antioxidant phase II enzymes and HSP70 help Z-LIG to protect cells from oxygen–glucose deprivation–reoxygenation (OGD-Reoxy)-induced injury, intranasal delivery of LIG enhances protection against ischemic injury via Nrf2 and HSP70 signaling pathways [158,186].

Z-LIG pretreatment of adipose-derived stem cells (ADSCs) significantly improves the therapeutic efficacy of ADSCs transplantation. ADSCs have been reported to protect neurons by direct cell replacement and indirectly by releasing factors from their secretome, and ADSCs transplantation shows potential for treating neuronal diseases [187]. Chi and colleagues explored the potential of ADSCs transplantation in improving recovery after stroke and the effect of Z-LIG pretreatment of ADSCs on the therapeutic efficacy of ADSCs transplantation through an experimental design in which ischemic stroke mice were induced by brain surgery and combined with intracerebroventricular injection of transplanted stem cells. The results showed that ADSCs pretreated with a specific concentration of Z-LIG had a better therapeutic effect than those not pretreated with Z-LIG [188].

Z-LIG has also been found to have the potential to prevent and treat postoperative cognitive dysfunction (POCD). Sun et al. showed that Z-LIG promotes proliferation and neurogenesis in the hippocampus through activation of ERK1/2 in hippocampal neural stem cells (H-NSCs), thereby facilitating cognitive deficits after transient global cerebral ischemia and reperfusion (tGCI/R) injury [189] (Table 2).

### 7.2. Z-Ligustilide and Vascular Dementia

#### 7.2.1. Improvement of Cholinergic Activity

Z-LIG may ameliorate cognitive dysfunction caused by chronic cerebral hypoperfusion by modulating central cholinergic. Cholinergic neurotransmission is a key mechanism of the central cholinergic system and is critical for cognitive function [191]. Choline acetyltransferase (ChAT) is involved in acetylcholine (ACh) synthesis [192], while acetylcholinesterase (AChE) terminates ACh signaling [193]. Kuang et al. showed that Z-LIG treatment significantly improved cognitive dysfunction in rats subjected to permanent ligation of both common carotid arteries (2VO). This was related to the fact that Z-LIG significantly increased the activity of ChAT and inhibited the activity of AChE in ischemic brain tissues, suggesting that Z-LIG has a potential therapeutic effect on VaD and cerebrovascular insufficiency [194].

ChAT is produced in the cytosol and is mainly localized at axon terminals, where it catalyzes the synthesis of ACh from choline and acetyl coenzyme A. Synthesized ACh is packaged into synaptic vesicles, where it is stored by the vesicular acetylcholine transporter (VAChT). Thus, axon and dendritic integrity is critical for ChAT transport [192]. Under the observation of microtubule-associated protein 2 (MAP-2) antibody staining, the number of neuronal dendrites in the parietal cortex and hippocampal CA1 area of 2VO rats treated with Z-LIG for seven days was abundant, coherent, and well-ordered. The integrity was kept intact, which indicated that Z-LIG was effective in alleviating the damage of neuronal dendrites in rats with chronic cerebral hypoperfusion [195] and that this effect appeared to be related to the antioxidant activity of Z-LIG [190].

#### 7.2.2. Anti-Oxidation

Oxidative stress is an essential pathogenetic mechanism in VaD and always causes irreversible neuronal damage [196]. The improvement of cognitive impairment by Z-LIG often cannot be circumvented by exploring mechanisms to counteract oxidative stress. In the study by Peng et al., Z-LIG exerted an ameliorative effect on cognitive impairment in VaD rats superior to that of nimodipine. Consistent with previous findings, Z-LIG treatment ameliorated oxidative stress by increasing the activity of endogenous antioxidant enzymes such as SOD, GSH-Px, and CAT, decreasing malondialdehyde (MDA) levels, and reversing 2VO-induced dysfunction of the free radical system in brain tissue [194,197,198]. In addition, Z-LIG reduced homocysteine levels in the brain while attenuating oxidative stress damage in the brains of VaD rats. Follow-up studies suggest that Z-LIG may exert neuroprotective effects on the cerebral cortex of VaD rats by modulating the AMPK/SIRT1 pathway [197]. Silent information regulator factor 2-related enzyme 1 (SIRT1) is a member of the Sirtuins family, and SIRT1 activates various signaling molecules to prevent or improve cognitive function through antioxidant or anti-apoptotic effects [199]. Activated AMPK enhances SIRT1 activity and deacetylates SIRT1 downstream targets, promoting cell survival and slowing VaD progression [200,201]. Notably, this pathway may also have a regulatory role in the anti-apoptotic activity of Z-LIG [197].

#### 7.2.3. Anti-Neuronal Apoptosis

Z-LIG protects neurons in the cerebral cortex and hippocampus from damage by inhibiting neuronal apoptosis. Feng et al. first reported the effect of Z-LIG on the cortex. They investigated the protective effect of Z-LIG on the parietal cortex and hippocampus of rats in a chronic cerebral hypoperfusion model by expanding the time window of cerebral hypoperfusion in Z-LIG-treated 2VO rats. The results showed that Z-LIG significantly improved the spatial learning and memory abilities of 2VO rats by inhibiting neuronal apoptosis, and Peng and colleagues’ experiments also reached the same conclusion [195,197,198]. The exact mechanism of Z-LIG’s inhibition of neuronal apoptosis is being studied more closely, providing more possibilities for Z-LIG to become a new option for the clinical treatment of VaD.

Mammalian brain neuronal metabolism cannot be achieved without the involvement of iron, yet excess iron in brain neurons may trigger a cascading response of neuronal death [202]. Z-LIG inhibits OGD/R-induced brain iron accumulation. Z-LIG inhibited the increase of HIF-1α protein content and up-regulated the expression of the ferritin light chain in OGD/R-treated SH-SY5Y cells. Moreover, Z-LIG did not affect the expression of transferrin receptor 1 in SH-SY5Y cells while reversing the inhibition of ferroportin 1 expression by OGD/R, suggesting that Z-LIG was able to increase iron release from OGD/R-treated cells and had no effect on cellular iron uptake. Thus, Z-LIG may reduce cellular iron by up-regulating iron-release proteins, reducing iron-mediated apoptosis to some extent [203].

#### 7.2.4. Inhibition of Astrocyte Proliferation

Ischemic stroke-induced reactive astrocyte proliferation, glial scarring, and other associated inhibitory molecules impede the regeneration of neurons after neuronal injury in most adult mammalian brains [204]. Glial fibrillary acidic protein (GFAP) is widely used as a marker of astrocyte activation and proliferation. It accumulates in reactive astrocytes. The effect of Z-LIG on the number of GFAP-immunoreactive astrocytes in the parietal cortex and hippocampal dentate gyrus of 2VO rats was measured. It was found that Z-LIG significantly reduced the immunoreactivity of GFAP in the parietal cortex and the hippocampal dentate gyrus, which may be beneficial to the regeneration of ischemic neurons in the 2VO rats and contribute to the improvement of the cognitive function of 2VO rats [194,195].

#### 7.2.5. Inhibition of Endoplasmic Reticulum Stress

Endoplasmic reticulum (ER) stress is closely associated with learning and memory deficits in VaD [205]. Inositol-requiring enzyme 1 alpha (IRE1α) is an endoplasmic reticulum-resident protein essential for the unfolded protein response (UPR), and activation of IRE1α with other endoplasmic reticulum-anchored receptors during ER stress maintains intracellular homeostasis [206]. However, when the UPR exceeds the ER load, the transcription factor X-box binding protein-1 (XBP-1) can be translocated to the nucleus to activate C/EBP homologous protein (CHOP) [207]. Activation of SIRT1 downregulates the ER stress pathway and attenuates ER stress-induced cellular damage [208,209]. Based on this, Peng et al. continued to explore the neuroprotective effects of Z-LIG on VaD and its possible mechanisms, demonstrating that Z-LIG attenuates neuronal damage and ameliorates cognitive dysfunction by inhibiting activation of the IRE1α/XBP1s/CHOP pathway through activation of SIRT1. The results of this study revealed that ER stress was significantly activated in VaD rats, and Z-LIG inhibited the activation of the IRE1α/XBP1s/CHOP pathway. Meanwhile, applying the SIRT1 inhibitor in the OGD model verified that the inhibition of SIRT1 partially eliminated the inhibitory effect of Z-LIG on the IRE1α/XBP1s/CHOP pathway [198] (Table 3).

## 8. Conclusions and Prospects

**Figure 3:**
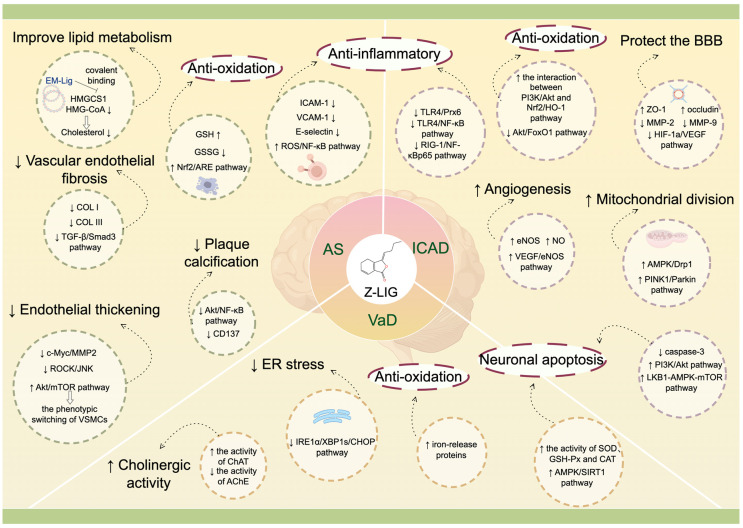
Molecular mechanisms of Z-ligustilide intervention in atherosclerosis complicating cerebrovascular disease. The picture is generated by Figdraw v.2.0.

Z-LIG mainly alleviates AS plaque formation and development by improving lipid metabolism, anti-oxidation, and anti-inflammation, up-regulation of endothelial protective factors, and inhibiting vascular endothelial fibrosis and excessive thickening. In the severe cerebrovascular complications of AS, Z-LIG plays an intervention role that mainly involves multiple mechanisms, such as anti-neuroinflammation, anti-oxidative stress, and anti-neuronal apoptosis. For ICAD, Z-LIG exerts pharmacological mechanisms such as protecting the BBB, promoting mitochondrial division, and promoting angiogenesis. Increasingly, molecular mechanisms are being discovered regarding Z-LIG intervention and treatment of atherosclerosis complicating cerebrovascular disease, including activation of the Nrf2/ARE signaling pathway to reduce cellular damage caused by oxidative stress; inhibition of the TGF-ß/Smad3 signaling pathway to block the transformation of vascular endothelial fibrosis phenotype; inhibition of the TLR4/NF-KB signaling pathway to reduce the inflammation-induced cerebral ischemic injury; regulation of caspase-3, PI3K/Akt, LKB1-AMPK-mTOR, and AMPK/SIRT1 signaling pathways to inhibit neuronal apoptosis; inhibition of HIF-1a/VEGF signaling pathway to protect BBB permeability; stimulation of PINK1/Parkin signaling pathway to promote mitochondrial division; activation of VEGF/eNOS signaling pathway to promote angiogenesis, etc. We also mentioned the correlation between EPO upregulation and neuroprotective effects of Z-LIG in Section 7.1.7. For VaD, the neuroprotective effect of Z-LIG is more favorable to improve cholinergic, inhibit astrocyte proliferation, and improve ER stress by inhibiting the IRE1a/XBP1s/CHOP signaling pathway (Figure 3).

Much evidence supports the underlying mechanisms by which diabetes exacerbates AS, including dyslipidemia, oxidative stress, and inflammation [210,211,212]. In response to diabetes mellitus, Z-LIG has also shown surprising pharmacological activity in lowering blood glucose. Previous studies have reported that LIG-rich total lactones may play an antidiabetic role by inhibiting islet cell apoptosis and regulating lipid metabolism disorders to improve glucose intolerance and dyslipidemia in HFD-fed and STZ-induced type 2 diabetic mice [213]. Furthermore, Z-LIG is able to attenuate insulin resistance and lipid accumulation in diabetic rats by activating the AMPK pathway and has a protective effect on diabetes-induced liver and kidney injury [214]. There is no doubt that Z-LIG is a drug with extensive research promise for the treatment of AS.

Safety research on Z-LIG is worth paying attention to. There are no studies on the toxic side effects of Z-LIG in in vivo experiments, but it has been reported that Z-LIG has a multi-organ protective effect [215,216,217,218]. However, in the in vitro experiments, cytotoxicity may occur beyond a specific concentration of Z-LIG. Also, the duration of action of Z-LIG may influence the outcome of its intervention to some extent. This effect may be connected with the absorption and metabolism of Z-LIG in vivo. Therefore, the appropriate concentration range and duration of action need to be mapped before conducting Z-LIG-related pharmacological experiments. In conclusion, Z-LIG may act as a relatively safe natural small molecule to fulfill its potential pharmacological effects, although the in vivo toxicity of Z-LIG demands more in-depth investigations.

Exploring alternatives to different routes of administration and developing new formulations are strategies that have been ongoing to address the low bioavailability and solubility of Z-LIG. Intranasal administration has effectively improved Z-LIG’s bioavailability by avoiding first-pass effects in the gut and liver and bypassing the BBB, resulting in rapid and broad CNS penetration. Z-LIG nanoemulsion effectively improves its solubility with more uniform and reproducible oral bioavailability [219].

In addition, the various available Z-LIG-containing materials inside and outside of organisms, including crude plant material, crude plant extracts, enriched fractions, and different purification levels of Z-LIG, showed very different degrees of degradation regarding abundance levels and chemical species diversity [220]. Thus, the synthesis and structural modification of Z-LIG need to be investigated. The other aspect, which has received even less attention, is that the currently observed biological activity of Z-LIG may be generated by its derivatives or degradation products. This means that monitoring reactivity, instability, and degradation chemistry is critical to understanding the biosignature of Z-LIG and any plant product containing or derived from it. This is an essential endeavor for future chemical and biological studies of Z-LIG and its formulations and will also provide new ideas for developing Z-LIG in relation to expanding the diseases it treats.

As a potential therapeutic agent for AS and its cerebrovascular complications, LIG extracted from virgin plant material exhibits unstable and easily degradable properties. Thus, the stability of LIG is also an urgent problem to overcome. Current response methods include applying self-microemulsifying drug delivery system (SMEDDS) [221], LIG inclusion with β-cyclodextrin [222], LIG liposome [223], and the addition of stabilizers nitrogen and fluorocarbon gas [224]. As E-LIG is more unstable than Z-LIG, the study of the E-type and its metabolism has been extremely limited, and further studies on the mass balance and stability of LIG may be able to achieve a breakthrough in this dilemma. Related derivatives of LIG reflect better stability and safety in drug preparation and application. Still, its pharmacological activity has yet to be investigated [225,226,227,228]. Therefore, further structural modification, synthesis, and screening of LIG can provide more opportunities for the progress of LIG clinical applications.

In conclusion, despite the potential of Z-LIG as an effective potential drug for AS and its cerebrovascular complications, most of the pharmacological experimental results of Z-LIG have been obtained on laboratory models and a lack of standardized research. There is a gap in clinical trials, so the addition of multi-dose, large-sample-size clinical trials is needed for the results of the currently available studies.

## Figures and Tables

**Table 1:** Mechanisms of action of Z-ligustilide intervention in atherosclerosis.

Pharmacological Effect	References	Applications	Animal/Cell Lines Models	Concentration of Z-LIG Used	Results	Mechanism(s)	Cytotoxicity
Improve lipid metabolism	[20]	in vitro	HepG2 cells (oleic acid-induced)	0, 50, 100, 200 μM	cholesterol synthesis ↓; FATP5 ↓, DGAT2 ↓	Inhibit the expression of the transcript for FATP5 and DGAT2	/
	[21]	in vivo	Male ApoE^−/−^ mice (C57BL/6J) (HFD)	10 or 20 mg/kg/d	lipid accumulation ↓; EM-Lig binds to Cys129 of HMGCS1 ↑; the biotransformation of HMG-CoA ↓	Inhibit cholesterol biosynthesis through the irreversible binding of the metabolic intermediate of Z-LIG to Cys129 of HMGCS1	/
		in vitro	HepG2 cells	0.1, 1, 10 µM			
Anti-oxidation	[22]	in vivo	Male Ldlr^−/−^ mice (C57BL/6JNju) (HFD)	20 mg/kg/d, i.p., for 8 w	body weight ↓, TC ↓, TG ↓, LDL-C ↓, HDL-C ↑; plaque areas ↓; MDA ↓, 4-Hne ↓; SOD ↑, CAT ↑, GPX ↑; GSH ↑, GSSG ↓, GSH/GSSG ↑; unbound Nrf2- Keap1 complex ↑; Nrf2 and ARE-driven enzyme ↑	Alleviate oxidative stress via stimulating the Nrf2/ARE pathway and attenuate atherosclerotic plaque formation	/
		in vitro	The EA.hy926 cell line	0–100 µM, for 24 h			500–1000 µM Z-LIG for 24 h impairs the viability of EA.hy926 cells
Anti-inflammatory	[19]	in vitro	HUVECs	1, 3, or 10 μM, for 30 min	ICAM-1 ↓, VCAM-1 ↓, E-selectin ↓; phosphorylation of IκB-α ↓, NF-κB p65 ↓; ROS ↓	Suppress ICAM-1, VCAM-1, and E-selectin expression in HUVECs and inhibit activated NF-κB signaling pathway	Z-LIG at 10 µM and above for 24 h impairs the viability of HUVECs
Endothelial protective effect	[19]	in vitro	HUVECs	1, 3, or 10 μM, for 30 min	HO-1 ↑, Nrf2 ↑; NO ↑	Induce HO-1 expression through Nrf2 nuclear translocation and endothelial NO synthesis	/
Alleviate vascular endothelial fibrosis	[28]	ex vitro	MAECs	10^−4^, 10^−5^, 10^−6^ mol/L	the binding of SMAD3 to TGF-β receptor 1 ↓; phosphorylation and nuclear translocation of SMAD3 ↓; COLI and COLIII ↓	Block the mutual interaction of SMAD3 and TGF-β receptor 1 and suppress COL I and COL III expression	/
Anti-vascular endothelial pathologic thickening	[24]	ex vitro	VSMCs from thoracic aorta of male Wistar rats	0, 10, 20, 30, 40 μg/mL, for 48 h	ERK ↓, JNK ↓, p38 ↓; ROS ↓; cyclin D1 protein ↓, p21 ↑, p-pRb ↓	Inhibit vascular endothelial cell proliferation and cell cycle progression by reducing ROS production and inhibiting the MAPK pathway	/
	[25]	in vivo	SHR	/	c-Myc ↓; a-SMA ↓; blood pressure and blood lipid ↓	Inhibit c-Myc pathway and variation of phenotypic switching	/
	[26]	in vivo	SHR(HFD)	10, 20, 40 mg/kg/d, i.p., for 2 w	blood pressure and blood lipid ↓; the migration of VSMCs ↓; c-Myc ↓; a-SMA ↓, MMP2 ↓; ROCK ↓, JNK ↓	Inhibit migration of VSMCs by suppressing c-Myc/MMP 2 and ROCK/JNK signaling pathways	Z-LIG at 10 µM and above for 6/12/24/48 h impairs the viability of rat VSMCs
	[27]	in vitro	Primary SHR VSMCs	0, 2.5, 5, 10, 20, 40 µg/mL, for 6/12/24/48 h	LC3-II ↓, ULK1 ↓, Beclin-1 proteins ↓; LC3-II/LC3 ↓, p-Akt ↑, p-mTOR ↑; NO ↑; ROS ↓; Ca^2+^ ↓	Inhibit A7r5 cell autophagy via the Akt/mTOR signaling pathway, reduce the concentrations of ROS and Ca^2+^, and upregulate the NO in A7r5 cells	Z-LIG at 10 µM and above for 6/12/24/48 h impairs the viability of rat VSMCs
			A7r5 cells (Ang II-induced)	0, 2.5, 5, 10 μg/mL, for 1 h			Z-LIG at 10 µM and above for 6/12/24 h impairs the viability of A7r5 cells
Anti-plaque calcification	[23]	in vitro	MAECs; HEK 293	10^−4^, 10^−5^, 10^−6^ mol/L	CD137 ↓; AP-1 ↓	Suppress the expression of CD137 by inhibiting AP-1 and Akt/NF-κB signaling pathways.	/

**Table 2:** Mechanisms of action of Z-ligustilide in the treatment of ischemic stroke and transient ischemic attack.

Pharmacological Effect	References	Applications	Animal/Cell Lines Models	Concentration of Z-LIG Used	Results	Mechanism(s)	Cytotoxicity
Anti-neuroinflammatory	[29]	in vivo	SD rats (MCAO)	20, 40 mg/kg/d, i.p.	Infarct volume ↓, neurological deficit score ↓; NeuN ↓, GFAP ↓, Iba-1 ↓, MPO ↓, CD3 ↓; TNF-α ↑, IL-1β ↑, ICAM-1 ↑, MMP 9 ↑, IFN-γ ↑, IL-17 ↑; IL-10 ↑; Prx 5 ↓, Prx 6 ↓; TLR4 ↓; phosphorylation of ERK 1/2 ↓; NF-κB ↓	Inhibit TLR4/Prx6 signaling	/
	[124]	in vitro	The murine macrophage-like cell line RAW264.7	5, 10, 20 μM, for 1 h	NO ↓, TNF-α ↓, IL-6 ↓; TLR4 ↓, iNOS ↓, NF-κB activation in the Prx-stimulated cells ↓	Inhibit Prxs, Prx1, Prx2, and Prx4-induced activation of the TLR4/NF-κB pathway in macrophages	Co-treatment of 5 to 20 μM Z-LIG with 20 nM Prx1, Prx2, or Prx4 did not impair macrophage viability
	[128]	ex vitro	Primary mixed glial cultures prepared from postnatal day 1 SD rat	2.5, 5, 10, 20 µmol/L	NO ↓, TNF-α ↓, IL-1β ↓, MCP-1 ↓; LPS-stimulated immunoreactivity of activated NF-κB, COX2, and iNOS ↓	Inhibit the NF-κB pathway in primary rat microglia	Pretreatment with 2.5 to 20 µmol/L Z-LIG for 1 h did not impair microglia viability
	[30]	in vivo	C57BL/6J mice (BCCAO)	30 mg/kg, i.p., at 0, 24, and 48 h after reperfusion	Neural injury ↓, neurological deficit score ↓; TNF-α ↓, IL-6 ↓, klotho ↑	Inhibit RIG-I/NF-κB p65 pathway and upregulate klotho expression	/
	[125]	in vitro	BV-2 cells (LPS-induced); PBMac (LPS-induced); Neuro-2a cells	50 μg/mL or 1, 5, 10, 25, 50 μM for 18 h (BV-2 cells); 50 μg/mL for 24 h (PBMac)	TNF-a and nitrite (BV-2 cells and PBMac) ↓; Neuro-2a cell death (BV-2 cells) ↓	Inhibit the production of proinflammatory mediators in murine BV-2 cells and PBMac	/
	[126]	in vivo	Adult male CD1 mice	200 μL/60 mg/kg, i.v.	TNF-α ↓, IL-1β ↓, IL-6 ↓; microglia activation ↓	Inhibit microglial activation and proinflammatory cytokine production	/
		ex vitro	BV2 cells; primary cultured microglial cells	1, 10, 50 μM (BV2 cells); 10 μM (primary cultured microglial cells)			
Anti-oxidation	[190]	in vitro	PC12 cells	0.1, 1, 2.5, or 5 μg/mL, for 24 h	Cell viability ↑; TAC ↑, ROS ↓	Provide significant protection against H_2_O_2_-induced cytotoxicity	/
	[32]	in vivo	Male ICR mice (FCI)	5 or 20 mg/kg, i.p.	The infarct volume ↓; MDA ↓; GSH-Px ↑; SOD ↑	Inhibit oxidative stress	/
	[31]	in vitro	Rat pheochromocytoma PC12 cells (OGD)	5 or 50 μM	Nrf2 ↑, HO-1 ↑; GSH ↓, SH ↓; the sequential phosphorylation of Akt kinase and GSK-3b ↑	Promote the cross-talks between redox signaling system, Nrf2 pathway, and PI3K/Akt pathway	Z-LIG treatment above 25 μM for 24 h impairs PC12 cell viability
	[30]	in vivo	C57BL/6J mice (BCCAO)	30 mg/kg, i.p., at 0, 24, and 48 h after reperfusion	Neural injury ↓, neurological deficit score ↓; MDA ↓, klotho ↑	Inhibit the Akt/FoxO1 pathway and upregulate klotho expression	/
Anti-neuronal apoptosis	[190]	in vitro	PC12 cells	0.1, 1, 2.5, or 5 μg/mL for 24 h	Cell viability ↑; Bax ↓, Caspase-3 ↓, Cytc ↓, Bcl-2 ↑	Provide significant protection against H_2_O_2_-induced cytotoxicity	/
	[32]	in vivo	Male ICR mice (FCI)	5 or 20 mg/kg, i.p.	The infarct volume ↓; Bcl-2 ↑, Bax ↓, caspase-3 ↓	Inhibit neuronal apoptosis	/
	[144]	in vivo	Adult male SD rats (MCAO)	20 mg/kg/d, i.p.	The infarct volume ↓, cerebral edema ↓; Ca^2+^ influx ↓, ROS ↓; the apoptosis ratio of hippocampal neurons ↓, Cytc ↓, Bax/Bcl-2 ↓; caspase-3 ↓	Activate the PI3K/Akt pathway, reduce calcium overload, and attenuate apoptosis	Z-LIG above 8 µM treatment for 24 h may impair hippocampal neuronal cell viability
		ex vitro	Hippocampus from newborn rat (OGD/R)	1, 2, 4 µM for 3 h			
	[152]	in vitro	PC12 cells (OGD/R)	1 × 10^−6^ M, for 3 h	PC12 cell proliferative activity ↑; PC12 cells apoptosis ↓; Bcl-2 ↑, Bax ↓; Beclin1 ↑, LC3-II ↑; autophagosomes ↑	Promote autophagy and protect PC12 cells from apoptosis via the LKB1-AMPK-mTOR signaling pathway	/
Improve blood–brain barrier permeability	[158]	in vivo	SD rats (MCAO)	15 mg/kg, i.n., for 3 d	Infarct volume ↓, BBB permeability ↓, brain edema ↓; the loss of collagen IV, occludin, and ZO-1 ↓; MMP-2 ↓, MMP-9 ↓	Enhance protection against ischemic injury by improving BBB permeability	/
	[154]	ex vitro	BMEC(OGD); astrocyte isolated from SD rats	5, 10, 20 μM, for 24 h	OGD-induced injury ↓; TEER values ↑, occludin ↑, ZO-1 ↑; HIF-1a ↓, VEGF ↓	Improve BBB permeability by protecting TJ proteins and inhibiting the HIF-1a/VEGF pathway	Z-LIG above 40 µM treatment may impair BMEC and astrocyte viability
Promote mitochondrial division	[166]	in vivo	SD rat (MCAO/R)	10 or 20 mg/kg	The neuronal damage ↓; Tomm20 ↓, COX4I1 ↓, LC3II/LC3I ↑, p62 ↓, PINK1 ↑, Parkin ↑; the colocalization of Tomm20 and LC3 ↑; ROS ↓; Na^+^-K^+^-ATPase ↑	Activate mitophagy mediated by PINK1/Parkin pathway	/
		in vitro	HT-22 cells (OGD/R)	20 μM			
	[169]	in vivo	SD rat (MCAO/R)	20 mg/kg/d	ROS ↓, Ca^2+^ ↓; MMP ↑; ATP ↑; Drp1 ↑, Fis1 ↑, LC3II/LC3I ↑, p62 ↓; AMPK phosphorylation ↑; cleaved-caspase-3 ↓	Promote Drp1-mediated mitochondrial fission via activation of AMPK	/
		in vitro	HT-22 cells (OGD/R)	20 μM			
Promote angiogenesis	[172]	in vivo	C57/BL6 mice (MCAO)	5, 10, and 20 mg/kg/d i.g./20 μM	Proliferation, migration, and tube formation of bEnd.3 ↑, the wound width ↑, infarct volume ↓, neurological function ↑; CD31^+^/BrdU^+^ cells ↑; VEGF ↑, eNOS ↑	Promote angiogenesis through local activation of the VEGF/eNOS pathway	/
		in vitro	bEnd.3 cells (OGD)				
Other effect	[158]	in vivo	SD rats (MCAO)	15 mg/kg, i.n., for 3 d	NQO1 ↑, HSP70 ↑	Enhance protection against ischemic injury via Nrf2 and HSP70 signaling pathways	/
	[186]	in vivo	Sprague–Dawley male rats (MCAO/R)	7.5, 15, or 30 mg/kg, i.n.	Infarct volume ↓, neurological function ↑; PC12 cells ↑; HSP70 ↑; phosphorylation of ERK1, 2 ↑, p38q ↑, JNK1, 2 ↑; ubiquitinated protein aggregation ↓	Induce protective HSP70 expression through activation of the MAPK pathway	/
		in vitro	PC12 cells (OGD-Reoxy); 293T cells (OGD-Reoxy)	5 µM for 16 h; 5 µM for 4 h			
	[188]	in vivo	BALB/c mice (MCAO)	30 or 90 mg/kg, s.c.	Nurr1 ↑, BDNF ↑, CXCR4 ↑, SDF1ab ↑; IL-6 ↓, IL-8 ↓; behavioral recovery ↑; brain cells from apoptosis ↓	Pretreatment of ADSCs with LIG improves the therapeutic efficacy of ADSCs transplantation	Treatment with 40 μg/mL Z-LIG for 24 h or 10 and 20 μg/mL Z-LIG for 48 h impair ADSCs viability
		ex vitro	ADSCs	0.625, 1.25, 2.5, and 5 μg/mL for 24 h			
	[189]	in vivo	Male C57/BL mice (tGCI/R)	80 mg/kg, i.g., for 1 month	Cognitive dysfunction ↓, Syp ↑, Psd-95 ↑, Gap-43 ↑, Syn-IIa ↑; hippocampal neuron damage ↓; hippocampal NSCs proliferation and neurogenesis ↑; the phosphorylation of ERK1/2 ↑, β-catenin ↓, NICD ↓, TLR4 ↓, NF-κB ↓;	Activate ERK1/2 in H-NSCs to promote their proliferation and neurogenesis in the hippocampus	Compared with H-NSCs exposed to 10 μM Z-LIG, the survival rate of H-NSCs exposed to 20 μM Z-LIG decreased by about 50%
		ex vitro	H-NSCs isolated from the hippocampus of 2-week-old mice	1.25, 2.5, 5, 10, and 20 μM			
	[181]	in vivo	SD rats (MCAO)	20, 40, or 80 mg·kg^−1^, i.g., for 3 and 0.5 h	Neurological deficit score ↓, infarct volume ↓; EPO ↑, RTP801 ↓; the cell viabilities ↑, LDH ↓,	Promote EPO transcription via an ERK signaling pathway and inhibit RTP801 expression	Pre-treatment of Z-LIG above 5 µM for 2 h impairs the viability of neurons in the mouse cerebral cortex
		ex vitro	Cortical neurons obtained from 15–16-day-old mouse embryos (OGD)	0, 0.625, 1.25, 2.5, 5, 10, or 20 mmol·L^−1^, for 2 h			

**Table 3:** Mechanisms of action of Z-ligustilide in the treatment of vascular dementia.

References	Pharmacological Effect	Applications	Animal/Cell Lines Models	Concentration of Z-LIG Used	Results	Mechanism(s)	Cytotoxicity
[194]	Improve cholinergic activity	in vivo	Wistar rats (2VO)	10 or 40 mg/kg, i.g., for 40 d	the activities of ChAT and AChE ↓	Improve cholinergic activity	/
	Anti-oxidation				MDA ↓, SOD ↑	Reversal of 2VO-induced dysfunction of the free radical system	
	Inhibit astrocyte proliferation				GFAP ↓, the pyramidal cells in CA1 regions ↑	Reduces GFAP-positive astrocytes in the hippocampal dentate gyrus	
[195]	Improve cholinergic activity	in vivo	SD rats (2VO)	80 mg/kg/d, i.g., for 7 d	MAP-2 ↑; GFAP ↓	Maintain dendritic integrity	/
	Anti-neuronal apoptosis				caspase-3 ↓	Inhibit neuronal apoptosis in the parietal cortex and hippocampus	
	Inhibit astrocyte proliferation				neuronal apoptosis ↓, caspase-3 ↓; MAP-2 ↑; GFAP ↓	Inhibit glial cell proliferation in the parietal cortex and hippocampus	
[197]	Anti-oxidation	in vivo	SD rats (2VO)	20 or 40 mg/kg, p.o., for 4 w	MDA ↓, SOD ↑, GSH-Px ↑; P-AMPK ↑, SIRT1 ↑	Reversal of 2VO-induced dysfunction of the free radical system via AMPK/SIRT1 pathway in VaD rats	/
	Anti-neuronal apoptosis				Bax ↓, caspase-3 ↓, Bcl-2 ↑, Nissl bodies ↑	Reduce neurodegeneration in VaD rats	
[198]	Anti-oxidation	in vivo and in vitro	SD rats (2VO); PC12 cells (OGD)	20 or 40 mg/kg, p.o.; 80 μM	MDA ↓, CAT ↑, SOD ↑, GSH-Px ↑	Reversal of 2VO-induced dysfunction of the free radical system	/
	Anti-neuronal apoptosis				Bax ↓, Bcl-2 ↑, Nissl bodies ↑	Reduce neurodegeneration in VaD rats	
	Inhibit endoplasmic reticulum stress				SIRT1 ↑, BIP ↓, P-IRE1α ↓, XBP1s ↓, CHOP ↓, PDI ↑	Improve cognitive impairment by regulating the SIRT1/IRE1α/XBP1s/CHOP pathway	
[203]	Anti-neuronal apoptosis	in vitro	SH-SY5Y cells (OGD/R)	0, 2.5, 25, or 50 µm, for 24 h	HIF-1a ↓; ferroportin 1 ↑, ferritin light chain ↑	Promote cell iron release and iron corporation into ferritin	Z-LIG at 50 µM for 24 h impairs the viability of SH-SY5Y cells

## Abbreviations

AS, atherosclerosis; IS, ischemic stroke; TIA, transient ischemic attack; VaD, vascular dementia; Z-LIG, Z-ligustilide; LIG, ligustilide; E-LIG, E-ligustilide; BBB, blood–brain barrier; ECs, endothelial cells; CAMs, cell adhesion molecules; ECM, extracellular matrix; LDL, low-density lipoprotein; VSMCs, vascular smooth muscle cells; SMCs, smooth muscle cells; ICAD, intracranial atherosclerotic disease; VCI, vascular cognitive impairment; TG, triglycerides; TRL, TG-rich lipoproteins; LDL-C, low-density lipoprotein cholesterol; FATP5, fatty acid transporter protease 5; DGAT2, diglyceride acyltransferase 2; HMGCS1, 3-hydroxy-3-methylglutaryl coenzyme A synthetase 1; TC, total cholesterol; EM-Lig, 6,7-epoxyligustilide; AM-Lig, alkynyl-modified Lig; ROS, reactive oxygen species; MMPs, matrix metalloproteinases; Nrf2, nuclear factor erythroid 2-related factor 2; GSH, glutathione; GSSG, glutathione disulfide; t-BHP, tert-butyl hydroperoxide; ARE, antioxidant response element; Keap1, Kelch-like ECH-associated protein 1; NF-κB, nuclear factor kappa-B; TNF-α, tumor necrosis factor alpha; VCAM-1, vascular cell adhesion molecule-1; ICAM-1, intercellular adhesion molecule-1; E-selectin, endothelial-selectin; HUVECs, human umbilical vein endothelial cells; NO, nitric oxide; HO-1, heme oxygenase 1; TGF-β, transforming growth factor-β; SMAD3, SMAD family member 3; COL I, collagen I; COL III, collagen III; MAECs, mouse aortic endothelial cells; MAPK, mitogen-activated protein kinase; ROCK, Rho-associated kinase; JNK, c-Jun N-terminal kinase; AngII, angiotensin II; mTOR, mammalian target of rapamycin; Akt, protein kinase B; TNFRSF, tumor necrosis factor receptor superfamily; TLRs, Toll-like receptors; DAMPs, damage-associated molecular patterns; Prxs, peroxiredoxins; ERK, extracellular signal-regulated kinase; LPSs, lipopolysaccharides; RIG-I, retinoic acid inducible gene I; CA1, cornu ammonis 1; Cpu, caudate-putamen; BCCAO, bilateral common carotid occlusion; SOD, superoxide dismutase; GSH-Px, glutathione peroxidase; CAT, catalase; FCI, forebrain cerebral ischemia; PI3K, phosphoinositide 3-kinase; FoxO, forkhead box class O; Cytc, cytochrome c; Bcl-2, B-cell lymphoma-2; Bax, Bcl-2-Associated X-protein; I/R, ischemia-reperfusion; OGD, oxygen–glucose deprivation; OGD/R, oxygen–glucose deprivation/reperfusion; MCAO/R, middle cerebral artery occlusion and reperfusion; CNS, central nervous system; LC3, microtubule-associated protein light chain 3; LKB1, liver kinase B1; TJs, tight junctions; HIF-1a, hypoxia-inducible factor 1 alpha; HIF-1, hypoxia-inducible factor 1; VEGF, vascular endothelial-derived growth factor; AQP-4, aquaporin-4; ZO-1, zonula occludens-1; MMP-2, matrix metalloproteinase-2; MMP-9, matrix metalloproteinase-9; PINK1, PTEN-induced putative kinase 1; Mdivi-1, mitochondrial division inhibitor 1; CIRI, cerebral ischemia-reperfusion injury; Drp1, dynamin-related protein 1; Mff, mitochondrial fission factor; bEnd.3, brain endothelial cell line; eNOS, endothelial nitric oxide synthase; EPO, erythropoietin; EpoR, erythropoietin receptor; HSP70, heat shock protein 70; OGD-Reoxy, oxygen–glucose deprivation–reoxygenation; ADSCs, adipose-derived stem cells; POCD, postoperative cognitive dysfunction; H-NSCs, hippocampal neural stem cells; tGCI/R, transient global cerebral ischemia and reperfusion; ChAT, choline acetyltransferase; Ach, acetylcholine; AChE, acetylcholinesterase; 2VO, common carotid arteries; VAChT, vesicular acetylcholine transporter; MAP-2, microtubule-associated protein 2; MDA, malondialdehyde; SIRT1, silent information regulator factor 2-related enzyme 1; GFAP, glial fibrillary acidic protein; ER, endoplasmic reticulum; IRE1α, inositol-requiring enzyme 1 alpha; UPR, unfolded protein response; XBP-1, X-box binding protein-1; CHOP, C/EBP homologous protein; SMEDDS, self-microemulsifying drug delivery system; HFD, high-fat diet; MAECs, mouse aortic endothelial cells; HEK 293, human embryonic kidney 293 cells; SHR, spontaneously hypertensive rats; HMG-CoA, 3-hydroxy-3-methylglutaryl coenzyme A; HDL-C, high-density lipoprotein cholesterol; 4-Hne, 4-hydroxynonenal; GSSG, glutathione disulfide; IκBα, inhibitor of nuclear factor-κB α; ULK1, UNC-51-like kinase 1; MCAO, middle cerebral artery occlusion; BCCAO, bilateral common carotid occlusion; PBMac, human peripheral blood monocyte-derived macrophages; BMEC, brain microvascular endothelial cell; COX2, cyclooxygenase-2; MCP-1, monocyte chemoattractant protein-1; TAC, tacrolimus; NQO1, quinone oxidoreductase 1; BDNF, brain-derived neurotrophic factor; Nurr1, nuclear receptor related 1; Syp, synaptophysin; GAP-43, growth-associated protein-43; NICD, notch intracellular domain.
